# Pharmacokinetics of Isavuconazole in Critically Ill Patients Receiving Extracorporeal Membrane Oxygenation (ECMO) Support: A Prospective Exploratory Observational Study

**DOI:** 10.3390/pharmaceutics18091049

**Published:** 2026-08-24

**Authors:** Alba Escolà-Rodríguez, Elena Sandoval, Jorge Moisés, Adrián Téllez Santoyo, Albert Carramiñana, Jaime I. Sainz de Medrano, Cristina Espinosa, Carlos Roca, Marta Hernández Meneses, Sabina Herrera, Mercè Brunet Serra, Pedro Castro, Dolors Soy Muner, Carla Bastida

**Affiliations:** 1 Pharmacy Department, Division of Medicines, Hospital Clínic de Barcelona, 08036 Barcelona, Spain; aescola@clinic.cat; 2 Facultat de Medicina i Ciències de la Salut, Universitat de Barcelona, 08036 Barcelona, Spain; 3 Department of Cardiovascular Surgery, Hospital Clínic de Barcelona, 08036 Barcelona, Spain; 4 Respiratory Intensive and Intermediate Care Unit, Department of Respiratory Medicine, Hospital Clínic de Barcelona, 08036 Barcelona, Spain; 5 Institut d’Investigacions Biomèdiques August Pi i Sunyer (IDIBAPS), Universitat de Barcelona, 08036 Barcelona, Spain; 6 Medical Intensive Care Unit, Hospital Clínic de Barcelona, 08036 Barcelona, Spain; 7 Surgical-Trauma Intensive Care Unit, Department of Anesthesiology and Critical Care, Hospital Clínic de Barcelona, 08036 Barcelona, Spain; 8 Department of Biochemistry and Molecular Genetics, Pharmacology and Toxicology Unit, Hospital Clínic de Barcelona, 08036 Barcelona, Spain; 9 Acute Cardiac Care Section, Hospital Clínic de Barcelona, 08036 Barcelona, Spain; 10 Infectious Diseases Department, Hospital Clínic de Barcelona, 08036 Barcelona, Spain; 11 Departament de Farmacologia, Toxicologia i Química Terapèutica, Facultat de Farmàcia i Ciències de l’Alimentació, Universitat de Barcelona, 08028 Barcelona, Spain

**Keywords:** extracorporeal membrane oxygenation, intensive care unit, isavuconazole, pharmacokinetics, drug loss

## Abstract

**Background:** Isavuconazole, a broad-spectrum triazole antifungal, exhibits high lipophilicity and extensive plasma protein binding, properties that may predispose it to sequestration within extracorporeal membrane oxygenation (ECMO) circuits. This study aimed to characterize the pharmacokinetics (PK) of isavuconazole and to evaluate drug sequestration within the ECMO circuit in critically ill patients receiving ECMO support. **Methods:** We conducted a prospective, exploratory, single-center observational study including critically ill patients receiving ECMO (veno-venous (VV) or veno-arterial (VA)) and treated with intravenous isavuconazole. Serial blood samples were collected simultaneously from the patient’s arterial line and from pre- and post-membrane oxygenator sampling sites. Non-compartmental analysis was performed on arterial line samples to estimate PK measures, and concentration differences across sampling sites were analyzed to estimate circuit-related drug loss. PK/pharmacodynamic (PD) target attainment was assessed using established efficacy thresholds (AUC_0–24_/MIC ≥ 25 and Cmin > 2 mg/L). **Results:** A total of 41 plasma samples from 3 critically ill patients (2 VV-ECMO, 1 VA-ECMO) were included in the analysis. Limited, component-specific isavuconazole loss was observed in tubing and connectors (6.62% ± 20.8%, *p* = 0.294) and across the entire ECMO circuit (7.74% ± 20.2%, *p* = 0.211). Likewise, no relevant concentration difference was detected across the membrane oxygenator (0.849% ± 6.15%, *p* = 0.642). Interindividual variability was observed across PK parameters, particularly in measures of elimination and distribution. All patients achieved predefined PK/PD efficacy targets, with mean Cmin and AUC_0–24_/MIC of 3.07 ± 0.261 mg/L and 85.3 ± 4.03, respectively, and none exceeded the established toxicity threshold. **Conclusions:** Preliminary results showed variable concentration differences across ECMO sampling sites, with no consistent pattern of isavuconazole loss across the ECMO circuit under the conditions evaluated. All patients achieved predefined PK/PD efficacy targets using currently recommended dosing regimens; however, interindividual PK variability was observed, supporting the potential value of therapeutic drug monitoring (TDM) to guide individualized dosing decisions in this population. Larger population PK studies are warranted to further characterize determinants of isavuconazole exposure during ECMO support and refine evidence-based dosing strategies.

## 1. Introduction

Extracorporeal membrane oxygenation (ECMO) is an advanced form of mechanical cardiopulmonary support used in critically ill patients with refractory cardiac and/or respiratory failure [1]. Although ECMO has significantly improved survival in selected populations, its use introduces important challenges for pharmacotherapy. The extracorporeal circuit comprises several components, all of which increase the extracorporeal circulating volume and provide a large surface area for drug adsorption and sequestration, potentially altering drug pharmacokinetics (PK) [2,3,4]. These effects are particularly relevant for lipophilic (Log P ≥1) and highly protein-bound compounds (generally >80%), which may lead to altered drug exposure and suboptimal therapeutic concentrations [5,6]. Beyond ECMO-related effects, critical illness itself can substantially alter drug disposition through changes in protein binding, organ function, capillary permeability, systemic inflammation, fluid balance, and altered drug distribution [7].

Invasive fungal infections represent a significant source of morbidity and mortality in the intensive care unit (ICU), particularly among patients with severe respiratory failure and prolonged critical illness [8]. Because successful treatment of these infections depends on adequate antifungal exposure, understanding the factors contributing to PK variability is particularly important in critically ill patients.

Isavuconazole is a broad-spectrum triazole antifungal widely used for the treatment of invasive aspergillosis and mucormycosis [9]. Its favorable pharmacological characteristics, including linear PK, high oral bioavailability, and the absence of cyclodextrin in the intravenous formulation, have contributed to its increasing use in critically ill populations [10]. Nevertheless, isavuconazole is characterized by high lipophilicity (log P 3.6), extensive plasma protein binding (>99%), and a large volume of distribution (Vd) [11]. These physicochemical properties make isavuconazole theoretically prone to adsorption and sequestration within ECMO circuit components, potentially reducing systemic drug exposure [11].

Despite the increasing use of ECMO in critically ill patients, the impact of extracorporeal support on isavuconazole exposure remains poorly characterized. Available evidence is scarce and largely limited to case reports and small observational studies, with inconsistent findings. Some studies have reported no relevant differences in exposure across the oxygenator membrane [12], whereas others have reported subtherapeutic concentrations and marked interindividual variability [13,14,15]. Therefore, the clinical significance of ECMO-related alterations in isavuconazole PK remains uncertain [12,13,14,15].

This exploratory study aimed to characterize the PK of isavuconazole in critically ill patients receiving ECMO, with particular focus on drug sequestration within different components of the extracorporeal circuit, including the membrane oxygenator, tubing and connectors. This study provides preliminary *in vivo* PK data of isavuconazole in critically ill patients receiving ECMO.

## 2. Materials and Methods

### 2.1. Study Population

We conducted a prospective, exploratory, observational, single-center study across five different ICUs at Hospital Clínic Barcelona, a Spanish tertiary university hospital. The study was approved by the local Ethics Committee (HCB/2024/0574) and was conducted in compliance with the ethical principles outlined in the Declaration of Helsinki. Prior to enrollment, written informed consent was obtained from all participants or, when applicable, from their legally authorized representatives.

Patients were eligible if they were aged ≥16 years, admitted to the ICU, receiving veno-venous (VV) or veno-arterial (VA) ECMO support, and treated with isavuconazole. Exclusion criteria included pregnancy, hypersensitivity to the study drug or its excipients, and patients who had received massive blood transfusion (>50% of the estimated total blood volume within the previous 8 h) and/or plasma exchange in the 24 h prior to PK sampling.

### 2.2. Demographic and Clinical Data

Clinical and demographic data were extracted from the hospital’s electronic medical records. Data included: (i) demographic characteristics (age, sex, total body weight, height, body mass index [BMI]); (ii) laboratory parameters such as serum creatinine, creatinine clearance (CLCR) estimated with the Cockcroft–Gault formula [16], and serum albumin; (iii) severity of illness scores, including the Sequential Organ Failure Assessment (SOFA) score and the Acute Physiology and Chronic Health Evaluation II (APACHE II) score, both calculated at ICU admission; (iv) use of renal replacement therapy (RRT), including intermittent hemodialysis or continuous renal replacement therapy (CRRT); (v) ECMO-related parameters, including ECMO configuration [VV or VA], device type (Rotaflow or CardioHelp), fraction of delivered oxygen (FDO_2_), sweep gas flow, blood flow rate, centrifugal pump settings, days under ECMO support, oxygenator membrane age (days in use at sampling), and presence of fibrin or clots at the time of sampling; (vi) antifungal therapy details, including isavuconazole dose, dosing frequency, route of administration, and infusion time; (vii) infection-related information, including infection type and microbiological isolates; and (viii) clinical outcomes, such as ICU length of stay, total hospitalization duration, and mortality at discharge.

### 2.3. Antifungal Administration and Blood Sampling

Eligible subjects received isavuconazole administered as a 1-h intravenous infusion via a central venous catheter. Isavuconazole was prescribed at the discretion of the treating physician, following the Summary of Product Characteristics [17], expert recommendations, and current antimicrobial treatment guidelines [18], when appropriate.

**Figure 1:**
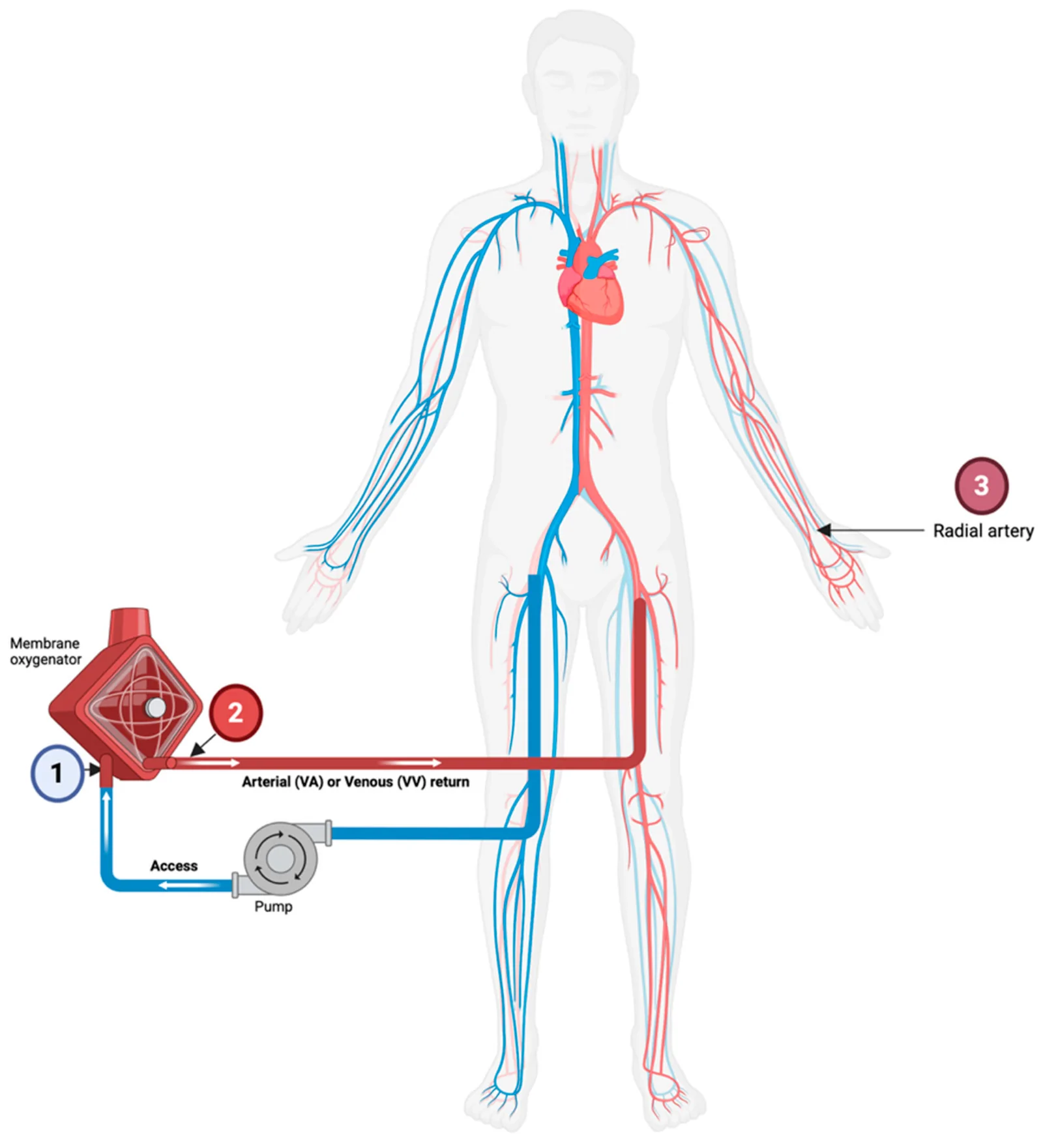
Sampling points used in the study: (1) pre-membrane oxygenator; (2) post-membrane oxygenator; and (3) the patient’s arterial line. Created in BioRender. Diaz-Ricart, M. (2026) https://BioRender.com/n2991kg.

Blood samples (4 mL collected in EDTA tubes) were collected from three sampling sites (pre-oxygenator, post-oxygenator and the arterial line) within a 5-min interval at each scheduled sampling time (Figure 1). This minimal interval reflects routine clinical practice, as all samples were collected sequentially by the same healthcare professional. Regarding the sampling sequence, pre-membrane samples were consistently collected before post-membrane samples. Arterial line samples were collected either before the pre-membrane sample or after the post-membrane sample, depending on the clinical workflow. All sampling times were precisely recorded.

Samples were collected over a complete dosing interval after completion of the isavuconazole loading regimen (200 mg every 8 h for six doses). Following the approved loading regimen, plasma concentrations were assumed to approximate steady-state exposure, as previously described for isavuconazole [17].

The sampling strategy was specifically designed to characterize isavuconazole PK over the full dosing interval and to enable the assessment of potential drug loss across the ECMO circuit, potentially reflecting drug sequestration within circuit components. For arterial line sampling, blood was collected at pre-dose (0 h) and at 1, 2, 4, 6, and 12 h post-infusion. For pre- and post-membrane oxygenator sampling, blood was obtained at pre-dose (0 h) and at 1, 2, and 6 h post-infusion. Immediately after collection, all samples were centrifuged, and plasma was separated and stored at −80 °C until further analysis.

### 2.4. Analytical Method

Isavuconazole total plasma concentrations were determined in the Laboratory of Pharmacology and Toxicology, CDB, Hospital Clinic Barcelona, using a previously validated ultra-high-performance liquid chromatography method coupled with tandem mass spectrometry (UHPLC-MS/MS) [19]. The analytical procedure was based on the ClinMass^®^ TDM Platform for antimycotics (RECIPE Chemicals + Instruments GmbH, Munich, Germany), using the manufacturer’s calibrators, quality controls and internal standard, with an in-house adapted sample preparation procedure validated according to the European Medicines Agency (EMA) Guideline on Bioanalytical Method Validation [20].

The calibration range was 0.481–10.8 mg/L, and the lower limit of quantification (LLOQ) was 0.481 mg/L. Quality control samples at two concentration levels were analyzed together with each analytical run. According to the laboratory validation data, intra- and inter-assay imprecision for isavuconazole was <3% and <7%, respectively, expressed as the coefficient of variation (CV).

### 2.5. Pharmacokinetic Analysis

PK measures were estimated using non-compartmental analysis (NCA). All analyses were performed using the actual recorded sampling times. As PK sampling was performed under steady-state conditions, pre-dose concentration (C_0_) was assumed to be equivalent to the concentration at the end of the dosing interval (C_24_) and was used as C_24_ for the calculation of the area under the plasma concentration–time curve from 0 to 24 h (AUC_0–24_) and the corresponding PK measures. Calculated PK measures included maximum plasma concentration (Cmax), time to maximum concentration (Tmax), AUC_0–24_, elimination rate constant (kel), total body clearance (CL), Vd, and elimination half-life (t_1/2_), based on plasma concentrations collected from the arterial line.

To evaluate potential drug loss across the ECMO circuit, differences in plasma concentrations between sampling sites were analyzed. Concentration differences were calculated at four scheduled sampling time points (pre-dose [0 h] and 1, 2, and 6 h after infusion) using paired plasma samples collected from the arterial line, pre-membrane, and post-membrane sampling sites. Specifically (i) the difference between pre-membrane and post-membrane concentrations (Cpre − Cpost) was used as an estimate of drug loss across the membrane oxygenator; (ii) the difference between post-membrane and arterial line concentrations (Cpost − Cart) was used to estimate drug loss in other ECMO circuit components, such as tubing and connectors; and (iii) the difference between pre-membrane and arterial line concentrations (Cpre − Cart) was considered a proxy for overall drug loss across the entire ECMO circuit.

In addition, the percentage of estimated drug loss was calculated for each comparison to normalize concentration differences relative to the corresponding reference sampling site, thereby providing a normalized estimate of concentration loss across each ECMO component and within the entire circuit. The percentage of drug loss was calculated as the concentration difference between sample sites divided by the corresponding reference concentration and multiplied by 100.

### 2.6. Dosing Adequacy and PK/PD Targets

The adequacy of isavuconazole exposure was assessed against predefined PK/pharmacodynamic (PD) efficacy targets. Target attainment was defined as achieving both a total trough concentration (Cmin) > 2 mg/L and an AUC_0–24_/MIC ratio ≥ 25 [21,22].

The EUCAST clinical breakpoint MIC for *Aspergillus* spp. (1 mg/L) was used as a surrogate MIC value to estimate PK/PD target attainment because isolate-specific MIC values for isavuconazole were not available [23]. Although a definitive PK/PD threshold associated with isavuconazole-related toxicity has not been established, trough concentrations >5 mg/L have been associated with an increased risk of toxicity in some reports [24].

### 2.7. Statistics

Descriptive and PK analyses were performed using R statistical software (version 4.6.0; R Foundation for Statistical Computing, Vienna, Austria) [25]. NCA was conducted using the PKNCA package in R (version 0.12.1; Denney et al., 2025) [26]. Categorical variables are presented as counts (percentage), while continuous data are expressed as mean ± standard deviation (SD) or median with interquartile range (IQR), as appropriate.

Pairwise comparisons between pre-membrane, post-membrane, and arterial line concentrations were performed using the paired *t*-test or Wilcoxon signed-rank test, depending on data normality. Given the exploratory nature of the study, no adjustment for multiple comparisons was performed. A *p*-value < 0.05 was considered statistically significant in all analyses.

## 3. Results

### 3.1. Baseline Clinical Characteristics

Three critically ill patients receiving ECMO were included, from whom a total of 41 blood samples were collected (17 arterial line, 12 pre-membrane, and 12 post-membrane samples). The median age was 75 years (IQR 67–76), and all patients were male (3/3, 100%). Two patients (66.7%) were supported with VV-ECMO for acute respiratory distress syndrome (ARDS), and one patient (33.3%) received VA-ECMO for cardiogenic shock. The median SOFA score was 11 (10–14), and the median APACHE II score was 24 (21–41).

At the time of sample collection, the median duration of ECMO support was 7 days (5–9), and the median oxygenator membrane age was 5 days (4–7). Evidence of oxygenator membrane clotting or fibrin deposition was observed in two patients (66.7%) on the day of sampling. All patients were under isavuconazole treatment for pulmonary aspergillosis, and *Aspergillus fumigatus* was isolated in all cases. The microbiological isolates were obtained from tracheal aspirate in one patient and from bronchoalveolar lavage in the other two patients. All patients received intravenous isavuconazole according to the approved dosing regimen. The median number of doses administered prior to sample collection was 8 (6–11).

Regarding hepatic function, two patients (66.7%) presented elevated total bilirubin concentrations, with values of 4.7 mg/dL and 3.6 mg/dL, respectively. Median AST and ALT concentrations were 57 U/L (27–144) and 21 U/L (7–58), respectively. All patients presented hypoalbuminemia, with median albumin concentrations of 21 g/L (20–27).

Two patients (66.7%) were under CRRT, which was integrated into the ECMO circuit in series. In all cases, CRRT was delivered as continuous veno-venous hemodialysis (CVVHD). The median effluent flow rate was 32.0 mL/kg/h (30.8–33.2). CRRT was maintained continuously without interruption during the PK sampling period. The patient who was not receiving CRRT had a serum creatinine of 0.48 mg/dL on the day of PK sampling, corresponding to an estimated creatinine clearance of 115.1 mL/min calculated using the Cockcroft–Gault equation.

Detailed demographic, clinical, and ECMO-related characteristics are summarized in Table 1.

### 3.2. Non-Compartmental Pharmacokinetic Analysis

NCA was performed using arterial line concentration–time data. The 12-h post-dose arterial sample was not available in one patient. Detailed PK measures, stratified by patient, are summarized in Table 2.

Interindividual differences in isavuconazole PK profiles were observed across the three patients (Table 2). The mean AUC_0–24_ was 85.3 ± 4.03 mg·h/L. Patient 1 showed the highest Cmax (8.99 mg/L) and the highest clearance (2.48 L/h), resulting in the lowest AUC_0–24_ (80.7 mg·h/L). In contrast, Patient 2 exhibited the longest elimination half-life (134 h) and the largest volume of distribution (438 L). Patient 3 displayed the lowest Cmax (4.82 mg/L) and the shortest elimination half-life (77.2 h). Overall, despite differences in individual PK measures, systemic exposure remained relatively consistent among the three patients.

### 3.3. Impact of ECMO Circuit on Isavuconazole Concentrations

**Figure 2:**
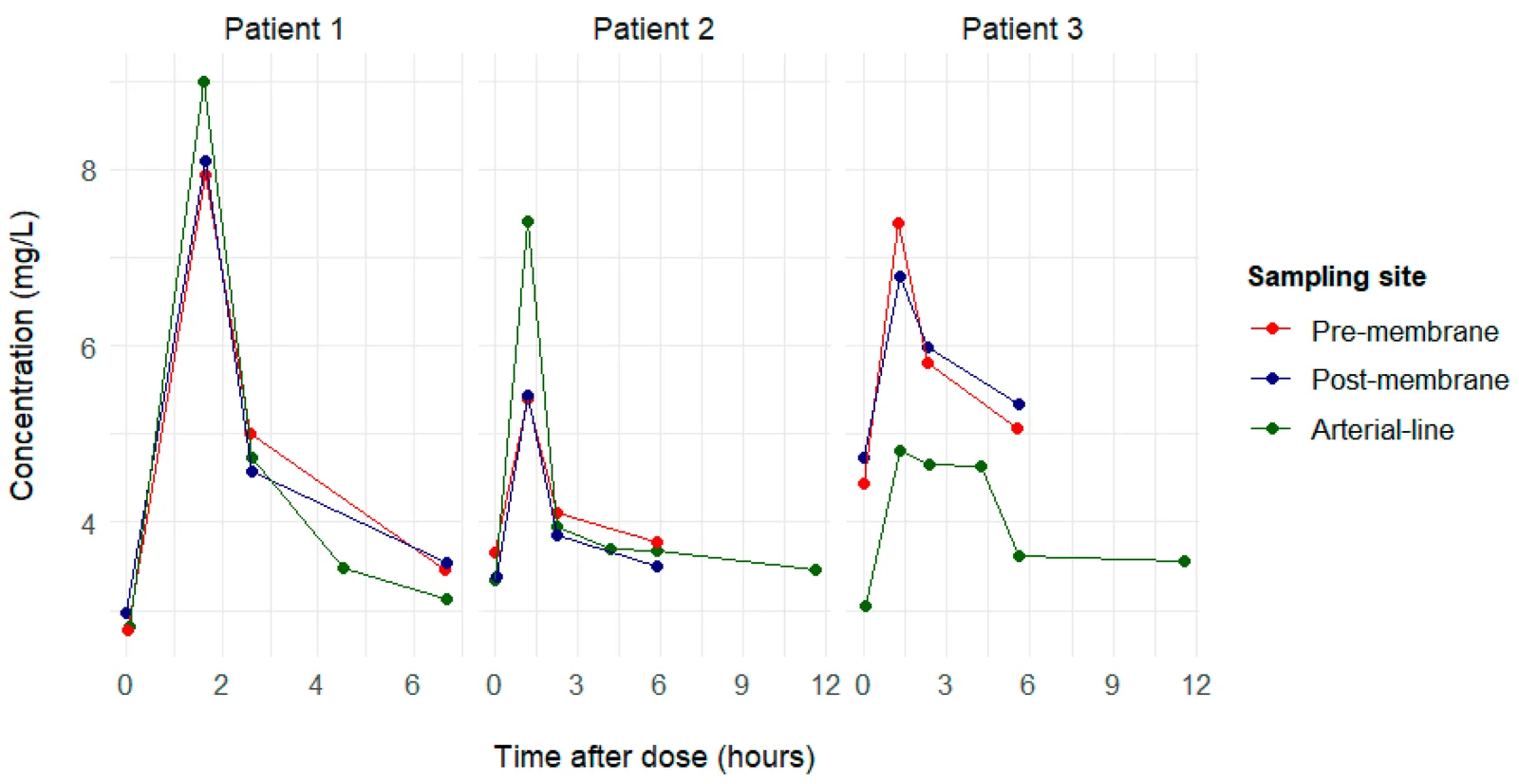
Individual plasma concentration–time profiles of isavuconazole, stratified by sampling site.

The analysis of isavuconazole concentrations across the three sampling sites (arterial line, pre-membrane, and post-membrane) was performed using 12 paired concentration measurements obtained from three patients (four paired measurements per patient). Small and variable concentration differences were observed between sampling sites, without a consistent pattern suggestive of isavuconazole loss across the ECMO circuit or its individual components. Results are presented as mean concentration differences (mg/L) and relative concentration differences (%) for the comparisons Cpre − Cpost, Cpost − Cart, and Cpre − Cart (Table 3). Individual plasma concentration–time profiles according to sampling site are illustrated in Figure 2.

Pre-membrane and post-membrane concentrations were generally higher than arterial line concentrations, resulting in small positive concentration differences between sampling sites. The mean relative concentration differences were 6.62 ± 20.8% (*p* = 0.294) between post-membrane and arterial line samples and 7.74 ± 20.2% (*p* = 0.211) between pre-membrane and arterial line samples.

Similarly, no relevant concentration difference was observed across the membrane oxygenator, with a mean relative concentration difference of 0.849 ± 6.15% (*p* = 0.642) and a mean concentration difference of 0.0500 ± 0.300 mg/L. However, given the limited sample size, these comparisons should be considered exploratory.

### 3.4. Target Attainment and Clinical Outcomes

Target attainment was assessed using arterial line concentrations. All three patients achieved the predefined PK/PD targets (Table 4), with individual AUC_0–24_/MIC values ranging from 80.7 to 88.3 and trough concentrations from 2.82 to 3.34 mg/L. None exceeded the proposed toxicity threshold.

Clinical outcomes are summarized in Table 1. The median hospital length of stay was 74 days (18–128), and the median ICU length of stay was 60 days (18–97). Overall mortality was 66.7% (2/3), including one patient receiving VV-ECMO and one patient receiving VA-ECMO.

## 4. Discussion

This prospective exploratory *in vivo* observational study provides preliminary insights into the PK of isavuconazole in critically ill patients undergoing ECMO support. To our knowledge, this is one of the few studies [12,27] that simultaneously evaluates isavuconazole plasma concentrations at three distinct sampling sites within the ECMO circuit—arterial line, pre-membrane, and post-membrane—in order to assess concentration differences across the ECMO circuit, explore the potential contribution of circuit-related drug sequestration and characterize isavuconazole PK during ECMO support.

While lifesaving, ECMO introduces substantial pharmacological complexity in critically ill patients due to profound pathophysiological alterations and the potential for drug sequestration within the extracorporeal circuit. Isavuconazole, given its lipophilicity and high plasma protein binding, carries physicochemical characteristics associated with susceptibility to ECMO sequestration. Nevertheless, our preliminary findings showed only small concentration differences across the ECMO circuit, with no consistent pattern of isavuconazole loss throughout the circuit. Concentration differences between pre- and post-membrane oxygenator samples were limited, suggesting that clinically relevant oxygenator-related sequestration is unlikely under the conditions evaluated.

All patients received standard dosing regimens, consisting of a loading dose of 200 mg every 8 h for 2 days followed by a maintenance dose of 200 mg once daily. Under these conditions, all patients achieved the predefined PK/PD efficacy targets, and no concentrations exceeded established toxicity thresholds. However, interindividual variability in isavuconazole PK was observed across the study population.

Beyond ECMO-related effects, interindividual variability appears to be largely driven by patient-specific factors. Isavuconazole is extensively metabolized via hepatic CYP3A4/5 and exhibits high plasma protein binding (>99%), making hepatic function and albumin concentration key determinants of exposure variability [28,29]. Reduced albumin concentrations may increase the unbound drug fraction, potentially altering tissue distribution and clearance. Additionally, systemic inflammation, capillary leak, and fluid shifts inherent to critical illness may further expand and destabilize the volume of distribution. Bolcato et al. demonstrated that both AST and albumin levels are independently associated with isavuconazole trough concentrations, with interindividual and intraindividual variability of 41.5% and 30.7%, respectively [30].

In our cohort, all patients presented with hypoalbuminemia, while two patients (Patients 1 and 2) exhibited significant hyperbilirubinemia (>1.2 mg/dL) alongside elevated AST levels (>40 U/L), suggestive of hepatic dysfunction in the context of critical illness. Notably, despite these shared clinical features, Patients 1 and 2 displayed distinct PK profiles, particularly in Vd and elimination half-life, suggesting that hepatic dysfunction and hypoalbuminemia do not fully account for the observed variability in isavuconazole disposition.

The PK profile of isavuconazole during ECMO has been previously described in small observational studies and case reports, with inconsistent findings that preclude definitive conclusions [12,13,14,15,27]. Kriegl et al. found no relevant differences in isavuconazole concentrations between pre- and post-membrane samples, concluding that the ECMO oxygenator did not significantly affect drug exposure in critically ill patients receiving VV- or VA-ECMO [12]. In contrast, Doménech-Moral et al. reported marked PK variability in critically ill patients on VV-ECMO, including evidence of significant drug loss across the membrane oxygenator in patients with recently replaced circuits [27]. Methodological differences between studies should be considered when interpreting these findings. Kriegl et al. evaluated isavuconazole concentrations following the first isavuconazole dose, whereas the present study and Doménech-Moral et al. assessed patients under steady-state conditions. Although pre- and post-membrane concentration comparisons allow direct evaluation of potential concentration changes across the oxygenator, cumulative exposure of the ECMO circuit to isavuconazole may influence the magnitude of drug–circuit interactions, as progressive saturation of binding sites within the circuit may reduce drug sequestration over time, particularly for lipophilic drugs with high protein binding. In our cohort, Patient 1, whose ECMO membrane had been replaced four days prior to sampling, exhibited the lowest systemic exposure. This finding is compatible with the hypothesis that newly replaced ECMO circuit components may contribute to transient drug sequestration through unsaturated binding sites, as previously described [27,31]. However, given the small sample size and the influence of multiple patient-specific factors on isavuconazole disposition, the contribution of membrane replacement to the observed exposure variability cannot be determined. In addition, other authors have reported subtherapeutic isavuconazole exposure with standard dosing during ECMO [13,14,15], requiring in some cases dose escalation to achieve adequate concentrations [13,14,15].

Regarding PK parameters, systemic CL in our study (2.35 ± 0.114 L/h) was consistent with previous population-based estimates from Desai et al. (2.36 L/h) [22] and Kovanda et al. (2.5 L/h) [32], but lower than the values reported by Martín-Cerezuela et al. (5.36 L/h) [10], potentially reflecting differences in illness severity, organ dysfunction, and extracorporeal support. Accordingly, the mean AUC_0–24_ (85.3 ± 4.03 mg·h/L) in our cohort was comparable to previously reported model-based estimates of 87.1 mg·h/L [32] and approximately 100 mg·h/L [22], suggesting relatively preserved systemic exposure despite ECMO support. Despite the variability in elimination parameters, overall exposure remained within the range reported in previous PK studies.

Overall, our findings suggest that the observed PK variability in this cohort may be more strongly influenced by the intrinsic heterogeneity of critically ill patients than by extracorporeal support itself. Dynamic changes in organ function, fluid status, plasma protein binding, and disease severity are likely more relevant determinants of isavuconazole disposition than ECMO-related effects. Despite this variability, all patients achieved adequate systemic exposure while receiving the currently recommended dosing regimen. These observations suggest that the currently recommended dosing regimen may provide adequate exposure in critically ill patients receiving ECMO support; however, given the small sample size and the heterogeneity of this population, larger studies are required before broader conclusions can be drawn. The magnitude and unpredictability of interindividual PK variability observed in this setting highlight that standard dosing alone may not reliably ensure adequate exposure in all ECMO-supported patients. In this context, therapeutic drug monitoring (TDM) emerges as an essential tool to guide individualized dosing decisions, detect subtherapeutic or supratherapeutic exposure early, and optimize clinical outcomes in ECMO-supported patients receiving isavuconazole. Future population PK studies will be critical to characterize interindividual and intraindividual variability, identify relevant covariates, and advance model-informed precision dosing (MIPD) strategies to generate robust, individualized isavuconazole dosing recommendations in ECMO-supported critically ill patients.

This study has several limitations. First, its single-center design and the small sample size may limit generalizability and preclude meaningful subgroup analyses, including comparisons between VV- and VA-ECMO configurations. Second, the heterogeneity of the cohort, including differences in ECMO configuration, concomitant CRRT use, hepatic function, membrane age, and severity of critical illness, may have contributed to the observed PK variability. Third, drug loss within the ECMO circuit was assessed using indirect estimations based on concentration differences between sampling sites rather than direct mass balance measurements. Comparisons involving arterial concentrations may therefore be influenced by patient-related factors such as drug distribution, metabolism, and clearance, particularly in the context of both VV- and VA-ECMO configurations. In VV-ECMO, arterial concentrations may include a component of recirculation within the ECMO circuit, whereas in VA-ECMO they may reflect the variable admixture of native cardiac output and ECMO blood flow. Importantly, the small sample size and observational design further limit the ability to distinguish circuit-related effects from patient-specific PK variability. Moreover, although concentration differences were evaluated using paired measurements, the small number of patients resulted in limited statistical power. Consequently, these analyses should be considered exploratory, and the corresponding *p*-values should be interpreted with caution, as non-significant results do not exclude the possibility of ECMO-related drug loss. Fourth, blood samples were collected quasi-simultaneously, with a maximum interval of up to 5 min between sampling sites, which may have introduced additional variability in measured concentrations. In addition, arterial line samples were not collected at a fixed point within the sampling sequence and could be obtained either before pre-membrane samples or after post-membrane samples; this may have introduced further variability in concentration measurements. However, this potential source of variability is considered limited in the case of isavuconazole, given its long elimination half-life and relatively stable PK profile. Fifth, two patients received CRRT integrated in series with the ECMO circuit, which may have introduced additional drug clearance and adsorption that could not be separately quantified. Furthermore, PK measures derived from the NCA should be interpreted cautiously. Although the sampling strategy covered a complete dosing interval under steady-state conditions by assuming that the pre-dose concentration (C_0_) was equivalent to the end-of-interval concentration (C_24_), the absence of a directly measured 24-h concentration represents a methodological assumption that may have introduced uncertainty in the estimation of AUC_0–24_ and related PK measures. In addition, the absence of the 12-h post-dose sample in one patient may have further limited the reliability of individual exposure and elimination estimates. Finally, due to the small sample size, no formal analysis of associations between PK/PD target attainment and clinical outcomes was possible.

Despite these limitations, the principal strength of this study lies in its simultaneous multi-site sampling approach across the ECMO circuit and intensive PK sampling, providing an exploratory characterization of isavuconazole PK and potential ECMO-related drug loss in a real-world critical care setting. Given the exploratory nature of the study and the small sample size, these findings should be interpreted cautiously and require confirmation in larger cohorts. Future studies incorporating population PK approaches will be essential to better characterize the determinants of isavuconazole exposure during ECMO support.

## 5. Conclusions

Our findings showed variable concentration differences across ECMO sampling sites, with no consistent pattern of isavuconazole loss across the ECMO circuit under the conditions evaluated. All patients achieved predefined PK/PD targets using currently recommended dosing regimens; however, these observations should be interpreted cautiously given the small sample size. The interindividual PK variability observed underscores the potential value of TDM to guide individualized dosing decisions and highlights the need for larger population PK studies to better characterize isavuconazole exposure and refine evidence-based dosing recommendations for critically ill patients receiving ECMO support.

## Figures and Tables

**Table 1:** Demographic and baseline clinical characteristics, ECMO parameters and patient outcomes stratified by patient.

	Patient 1	Patient 2	Patient 3
**Demographics and clinical characteristics**			
Age (years)	75	76	67
Sex	Male	Male	Male
Weight (kg)	73	65	54.5
Height (cm)	178	162	164
BMI (kg/m^2^)	23.0	24.8	20.3
CRRT (yes/no) CRRT modality	Yes CVVHD	Yes CVVHD	No -
Serum creatinine (mg/dL) *	-	-	0.48
CLCR (CG) (mL/min) *	-	-	115.1
Serum albumin (g/L)	20	21	27
Total bilirubin (mg/dL)	4.7	3.6	0.7
AST (U/L)	144	57	27
ALT (U/L)	58	7	21
SOFA ^†^	10	14	11
APACHE II ^†^	24	41	21
Vasopressors (yes/no)	Yes	Yes	Yes
**ECMO parameters**			
Type (VV/VA)	VV	VA	VV
Device type (Rotaflow/CardioHelp)	Rotaflow	CardioHelp	CardioHelp
FDO2 (%)	90	40	100
Sweep gas (L/min)	2	3	2
Blood flow (L/min)	4.2	3.6	3
Centrifugal pump (rpm)	3200	3600	2730
Days on ECMO	9	7	5
Oxygenator membrane replacement before PK sampling (yes/no)	Yes	No	No
Days of ECMO membrane	4	7	5
Clotting/fibrin deposition in ECMO membrane	Yes (clotting and fibrin deposition)	No	Yes (fibrin deposition)
**Antimicrobial therapy and clinical outcomes**			
Dose/Frequency	200 mg/8 h for 2 days, followed by 200 mg/24 h	200 mg/8 h for 2 days, followed by 200 mg/24 h	200 mg/8 h for 2 days, followed by 200 mg/24 h
Number of doses administered prior to PK sampling	6	11	8
Infection type	Pulmonary aspergillosis	Pulmonary aspergillosis	Pulmonary aspergillosis
Microbiological isolates	*Aspergillus fumigatus*	*Aspergillus fumigatus*	*Aspergillus fumigatus*
ICU length of stay (days)	18	97	60
Duration of hospitalization (days)	18	128	74
In-hospital mortality (yes/no)	Yes	Yes	No

Data were recorded on the day of pharmacokinetic (PK) sampling. ^†^ SOFA and APACHE II scores were calculated on ICU admission. * Calculated in patients without CRRT. ALT: alanine aminotransferase; APACHE II: Acute Physiology and Chronic Health Evaluation II score; AST: aspartate aminotransferase; BMI: Body Mass Index; CG: Cockcroft–Gault; CLCR: creatinine clearance; CRRT: Continuous Renal Replacement Therapy; CVVHD: continuous veno-venous hemodialysis; FDO2: fraction of delivered oxygen; ICU: Intensive Care Unit; SOFA: Sequential Organ Failure Assessment score; VA: veno-arterial ECMO; VV: veno-venous ECMO.

**Table 2:** Pharmacokinetic measures of isavuconazole stratified by patient.

Study Patient	Cmax (mg/L)	Tmax (h)	AUC_0–24_ (mg·h/L)	CL (L/h)	Vd (L)	Kel (h^−1^)	t_1/2_ (h)
Patient 1 [n = 6]	8.99	1.02	80.7	2.48	288	0.00860	80.6
Patient 2 [n = 7]	7.41	1.08	88.3	2.26	438	0.00517	134
Patient 3 [n = 7]	4.82	1.13	86.9	2.30	256	0.00898	77.2
Overall mean ± SD	7.07 ± 2.11	1.08 ± 0.0585	85.3 ± 4.03	2.35 ± 0.114	327 ± 97.1	0.00758 ± 0.00210	97.3 ± 31.9

Values in brackets [n = x] indicate the number of concentration–time points used for the non-compartmental analysis. AUC_0–24_: area under the plasma concentration–time curve from 0 to 24 h; CL: clearance; Cmax: maximum plasma concentration; Kel: elimination rate constant; t_1/2_: half-life; Tmax: time to maximum concentration; Vd: volume of distribution.

**Table 3:** Differences in isavuconazole concentrations between ECMO sampling sites.

	Cpre − Cpost	Cpre − Cart	Cpost − Cart
Concentration difference (mg/L)	0.0500 ± 0.300	0.387 ± 1.19	0.337 ± 1.17
Relative concentration difference (%)	0.849 ± 6.15 *p* = 0.642	7.74 ± 20.2 *p* = 0.211	6.62 ± 20.8 *p* = 0.294

Values are presented as mean ± SD. Cart: arterial line concentration; Cpost: post-membrane concentration; Cpre: pre-membrane concentration.

**Table 4:** Patient-Specific PK/PD Target Attainment.

	AUC_0–24_/MIC	Cmin (mg/L)
Patient 1	80.7	2.82
Patient 2	88.3	3.34
Patient 3	86.9	3.05
Mean ± SD	85.3 ± 4.03	3.07 ± 0.261

MIC = 1 mg/L was assumed for all patients. AUC_0–24_: area under the plasma concentration–time curve from 0 to 24 h; Cmin: trough plasma concentration at steady state (mg/L); MIC: minimum inhibitory concentration (mg/L).

## Data Availability

Data available on request. The data underlying this article will be shared on reasonable request to the corresponding authors due to ethical and privacy considerations.
